# Supplementary material for: MetaRibo-Seq measures translation in microbiomes
Source: Nat Commun. 2020 Jun 29;11:3268. doi: 10.1038/s41467-020-17081-z (PMC7324362; doi:10.1038/s41467-020-17081-z)
Supplement: Supplementary file 10 — Supplementary Data 7 [file 41467_2020_17081_MOESM10_ESM.zip › File2/Confidence_High_Taxonomy/156146_out.krona.html]

Javascript must be enabled to view this page.

members
magnitude
magnitudeUnassigned
count
unassigned
taxon
rank

156146\_out

11

2
superkingdom
11

11
phylum
1239

186801
class
11

186802
11
order

186803
11
family

33042

SRS065397\_contig\_number\_contig-100\_18512.61943SRS147139\_contig\_number\_7930SRS148784\_contig\_number\_contig-100\_22478.60846
11
genus
3

4
species

SRS012273\_contig\_number\_33569SRS012969\_contig\_number\_20766SRS017103\_contig\_number\_25500SRS063040\_contig\_number\_16673
33043

species
1

SRS015579\_contig\_number\_610
2293088

1896997
2
species

SRS024331\_contig\_number\_20970SRS104400\_contig\_number\_35564

2293090
1
species

SRS020869\_contig\_number\_20008
